# Detection of MCPG metabolites in horses with atypical myopathy

**DOI:** 10.1371/journal.pone.0211698

**Published:** 2019-02-05

**Authors:** Mandy Bochnia, Johannes Sander, Joerg Ziegler, Michael Terhardt, Stefanie Sander, Nils Janzen, Jessika-M. V. Cavalleri, Aleksandra Zuraw, Monika Wensch-Dorendorf, Annette Zeyner

**Affiliations:** 1 Group Animal Nutrition, Institute of Agricultural and Nutritional Sciences, Martin Luther University Halle-Wittenberg, Halle (Saale), Germany; 2 Screening-Labor Hannover, Hannover, Germany; 3 Department of Molecular Signal Processing, Leibniz Institute of Plant Biochemistry, Halle (Saale), Germany; 4 Department of Clinical Chemistry, Hanover Medical School, Hannover, Germany; 5 Clinic for Horses, University of Veterinary Medicine Hannover, Foundation, Hannover, Germany; 6 Definiens AG, München, Germany; 7 Group Biometrics, Institute of Agricultural and nutritional Sciences, Martin Luther University Halle-Wittenberg, Halle (Saale), Germany; College of Agricultural Sciences, UNITED STATES

## Abstract

Atypical myopathy (AM) in horses is caused by ingestion of seeds of the *Acer* species (*Sapindaceae* family). Methylenecyclopropylacetyl-CoA (MCPA-CoA), derived from hypoglycin A (HGA), is currently the only active toxin in *Acer pseudoplatanus* or *Acer negundo* seeds related to AM outbreaks. However, seeds or arils of various *Sapindaceae* (e.g., ackee, lychee, mamoncillo, longan fruit) also contain methylenecyclopropylglycine (MCPG), which is a structural analogue of HGA that can cause hypoglycaemic encephalopathy in humans. The active poison formed from MCPG is methylenecyclopropylformyl-CoA (MCPF-CoA). MCPF-CoA and MCPA-CoA strongly inhibit enzymes that participate in β-oxidation and energy production from fat. The aim of our study was to investigate if MCPG is involved in *Acer* seed poisoning in horses. MCPG, as well as glycine and carnitine conjugates (MCPF-glycine, MCPF-carnitine), were quantified using high-performance liquid chromatography-tandem mass spectrometry of serum and urine from horses that had ingested *Acer pseudoplatanus* seeds and developed typical AM symptoms. The results were compared to those of healthy control horses. For comparison, HGA and its glycine and carnitine derivatives were also measured. Additionally, to assess the degree of enzyme inhibition of β-oxidation, several acyl glycines and acyl carnitines were included in the analysis. In addition to HGA and the specific toxic metabolites (MCPA-carnitine and MCPA-glycine), MCPG, MCPF-glycine and MCPF-carnitine were detected in the serum and urine of affected horses. Strong inhibition of β-oxidation was demonstrated by elevated concentrations of all acyl glycines and carnitines, but the highest correlations were observed between MCPF-carnitine and isobutyryl-carnitine (r = 0.93) as well as between MCPA- (and MCPF-) glycine and valeryl-glycine with r = 0.96 (and r = 0.87). As shown here, for biochemical analysis of atypical myopathy of horses, it is necessary to take MCPG and the corresponding metabolites into consideration.

## Introduction

Atypical myopathy (AM) of horses is a frequently fatal disease characterized by acute rhabdomyolysis in pastured horses that consume seeds of *Acer spp*. (e.g., *Acer pseudoplatanus*, *Acer negundo*) [1–5] that belong to the *Sapindaceae* family of plants. Very young horses are particularly affected [3, 4]. In human medicine, it is well known that fruits of *Sapindaceae* can be very poisonous [6–9]. Early and on-going studies of the chemical components of these plants have shown that seeds and arils of *Sapindaceae (*ackee, lychee, longan, mamoncillo fruits) may contain the toxins hypoglycin A (HGA) and the lower homologue of HGA, methylenecyclopropylglycine (MCPG) [7, 10–16]. For example, in 1976, cases of Jamaican vomiting sickness (JVS) were investigated and conclusively linked to HGA in ackee fruits [17]. In other *Sapindaceae*, HGA alone (longan fruit), and in combination with MCPG (lychee fruit), was isolated from the fruits [11, 18, 19]. However, not all members of the *Sapindaceae* family produce both toxins, which seems to be associated with the ripeness of the various fruits [20, 21]. The ingestion of ackee and lychee fruits led to the detection of the metabolic products of exposure to HGA and MCPG [16]. These specific urinary metabolites of HGA and MCPG are methylenecyclopropylacetyl-glycine (MCPA-glycine) and methylenecyclopropylformyl-glycine (MCPF-glycine), respectively.

A histological hallmark of acute *Acer* seed poisoning in horses is lipid storage myopathy in skeletal muscle and sometimes in the myocardium. Biochemically elevated activities of muscle enzymes such as creatine kinase (CK) are observed. Furthermore, typically high concentrations of a broad spectrum of acyl carnitines (e.g., butyryl-carnitine) and acyl glycines (e.g., valeryl-glycine) are a result of the interruption of fatty acid β-oxidation by the inhibition of acyl-CoA dehydrogenases and enoyl-CoA hydratases [1]. Considering the accumulation of a broad spectrum of acyl conjugates, AM has been compared to a human inborn error of metabolism called multiple acyl-CoA deficiency (MADD) [1]. However, while HGA is responsible for the inhibition of acyl-CoA-dehydrogenases catalysing the first of the 4 steps of the β-oxidation cascade, the active metabolite of MCPG, MCPF-CoA, is known to cause the inhibition of the second step, which is performed by the enoyl-CoA hydratases [22–24]. If it can be shown that the inhibition of enoyl-CoA hydratases caused by MCPG ingestion contributes to the development of the disease, AM can no longer be interpreted as just an induced form of MADD.

**Fig 1:**
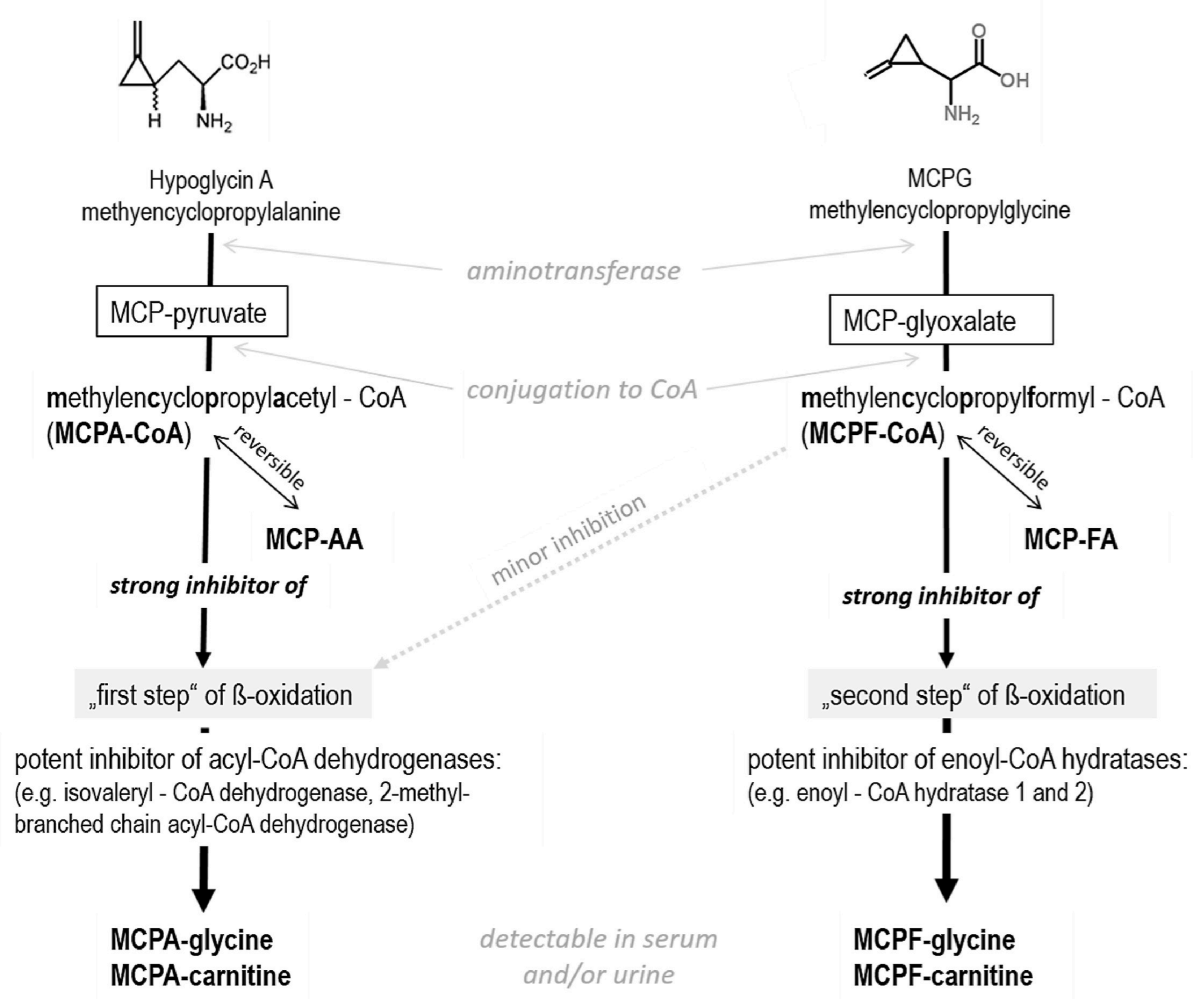
Metabolic pathway for MCPG and HGA after ingestion and possible mechanisms of excretion.

Recent research on *Sapindaceae* poisoning that resulted in atypical myopathy in horses only focused on HGA toxicity, while MCPG or conjugates of its metabolite methylenecyclopropylformate (MCPF), MCPF-glycine or MCPF-carnitine, have not yet been described in AM. However, several species of *Acer* have been reported to contain MCPG and/or HGA [12], which have both been documented as inducers of encephalopathy and hypoglycaemia in experiments conducted in rats [13, 19, 25–29]. Previous studies suggest that MCPG undergoes a similar metabolic pathway as HGA [13, 19, 22, 23, 30]. Although MCPG also inhibits the β-oxidation of fatty acids, it acts on a different step of the spiral degradation process (Fig 1). Therefore, the simultaneous action of the two homologues may increase the toxic effects.

To better understand the pathomechanism of *Acer* seed poisoning in horses, it is important to know to what extent each of the toxins, HGA and MCPG, is involved. Therefore, the objective of this study was to analyse serum and urine samples of horses affected by AM for the presence of MCPG, MCPF-glycine and MCPF-carnitine and to compare these results to those for HGA and its metabolites. In addition, we assessed the degree of enzyme inhibition by measuring the concentrations of several acyl glycines and acyl carnitines.

## Materials and methods

### Case selection

In autumn 2016 (November) and autumn 2017 (October–December), 14 horses from Germany (5 warmbloods, 1 cold-blooded horse, 3 heavy-warm-blooded horses, 3 Arabian horses, and 2 ponies; including 1 gelding, 5 stallions, and 7 mares; 1.0–2.0 y old, 24 h pasture turnout) were used as study subjects. All horses exhibited acute clinical signs of muscle pain and weakness. The farm veterinarians conducted the initial treatment and diagnosed AM based on the clinical signs and clinicopathological assessment. Eight horses were admitted to the equine clinic and Research Centre of Medical Technology and Biotechnology (Bad Langensalza, Germany), and three horses were admitted to the equine clinic at the Department of Large Animal Medicine (Faculty of Veterinary Medicine in Leipzig, Germany) for intensive care where they died or were euthanized (anaesthesia: 0.12 mg/kg body weight (bwt) romifidine; 0.2 mg/kg bwt diazepam; 2.0 mg/kg bwt ketamine; euthanasia: 5 ml/50 kg bwt T61). The last three horses were treated on the farm but died or were euthanized within a maximum of 2 d after the onset of disease (anaesthesia: 1.1 mg/kg bwt xylazine; 2.0 mg/kg bwt ketamine; euthanasia: 5 ml/50 kg bwt T61). The horses were kept on pasture, and clinical signs included the sudden onset of indicators such as the rapid progression of acute rhabdomyolysis with myoglobinuria, stiffness, trembling and sweating, weakness, recumbency, depression, and unexpected death. Laboratory tests showed very high activity of creatine kinase, lactate dehydrogenase and aspartate amino transferase. None of the 14 horses included in our investigation survived. Data of the diseased horses in this study are summarized in Table 1.

**Table 1:** Specific characteristics (breed, sex, age and creatinine kinase activity) of AM affected horses.

AM Horse	Breed	Sex	Age	CK^1^ [U/L]
1	Arabian horse	Stallion	1	655,022
2	Arabian horse	Mare	1	925,365
3	heavy WB	Mare	1.5	> 2,036*
4	heavy WB	Mare	1.5	> 2,036*
5	Arabian horse	Stallion	1	266,616
6	Cold-blooded horse	Stallion	2	98,795
7	WB	Stallion	2	> 2,036*
8	WB	Stallion	2	96,135
9	Pony	Gelding	1.5	73,500
10	Welsh Pony	Mare	1.5	1,682,500
11	heavy WB	Mare	1.5	681,000
12	WB	Mare	1	> 100,000*
13	WB	Stallion	1	> 20,000*
14	WB	Mare	1	382,477

AM, atypical myopathy

^1^ creatine kinase in serum samples

*not finally determined with serial dilution

### Blood and urine samples

Serum samples and ante-mortem urine samples were collected from all affected horses during the disease by qualified veterinarians through their routine practice using a framework of official programmes (blood sampling from the jugular vein, urine sampling with a catheter). For an individual horse both body fluids were obtained on the same day.

Samples were collected in order to perform diagnostic analyses during the course of the AM (e.g., measuring activities of creatine kinase and lactate dehydrogenase as well as several liver enzymes). The horse owners agreed verbally that residual material might be used for scientific research.

Additionally, serum of 12 horses that were clinically healthy and did not have access to *Acer pseudoplatanus* seeds were included in the study and served as controls. Urine samples of those control horses (n = 6) were voluntarily collected as free catch urine by the owners. All samples were kept frozen at—18°C until analysis.

### Seed samples

The affected horses originated from six different pastures. However, all affected horses had access to *Acer pseudoplatanus* seeds, and the HGA content in the seeds was determined. Five affected pastures were visited by the farm veterinarian directly after the occurrence of AM to collect seed samples. The collection was executed on private land, and the owners gave permission to conduct the study on their land. The focus was directed towards *Acer spp*., especially sycamore maple trees (*Acer pseudoplatanus*), to determine the presence of the seeds and availability to the horses. Analysis of the HGA content in the seeds was performed according to Bochnia et al. (2015) [31].

### Ethics statement

Animal Care and Use Committee approval was not requested for this study because no animals were handled specifically for this experiment. Blood collection was done by an experienced veterinarian from the jugular vein either at pasture on the farm or on admission at the clinics. The detection of the content of HGA and MCPA- and MCPF-conjugates was conducted with the owner's consent. Therefore, legal restrictions do not apply, as they are waived in the case of non-experimental procedures and routine veterinary practices in patients and companion animals (i.e., no laboratory animals were used).

### Reagents

MCPG was provided as a mixture of diastereomers under the chemical name (2S)-amino[(1S)-2-methylenecyclopropyl]acetic acid or 2-amino-3-(2-methylidenecyclopropyl)propanoic acid, unlabelled and [^13^C_2_^15^N]-labelled and was a generous gift of M. Carter, Centers for Disease Control, Atlanta, USA (contracted from IsoScience, King of Prussia, PA, USA). The purity of the unlabelled material was ≥97% and that of the isotopically labelled form was ≥99.3%. Both isomers made up approximately 50% of the total amount of the mixture.

The MCPF-glycine used was 97% pure, and the internal standard (97% pure) was ^13^C_2_^14^N MCPF-glycine (IsoScience, King of Prussia, PA, USA). MCPF-carnitine was quantified using d7-butyryl carnitine (ten Brink, Amsterdam, The Netherlands) as an internal standard, which was also used for quantifying butyryl carnitine.

For measurement of MCPA-glycine, this substance and ^13^C_2_^14^N MCPA-glycine were available at 97% purity (IsoScience), and the internal standard for the respective carnitine was d3-octanoyl carnitine (ten Brink). We used d3-leucine (Cambridge Isotope Laboratories, Teddington, UK) for HGA and d3-valeryl glycine for valeryl glycine (ten Brink). The HGA was purchased as an 85% pure substance (Toronto Research Chemicals, Toronto, Canada). General laboratory reagents were analytical grade of the best quality available.

### Analysis MCPG

**Fig 2:**
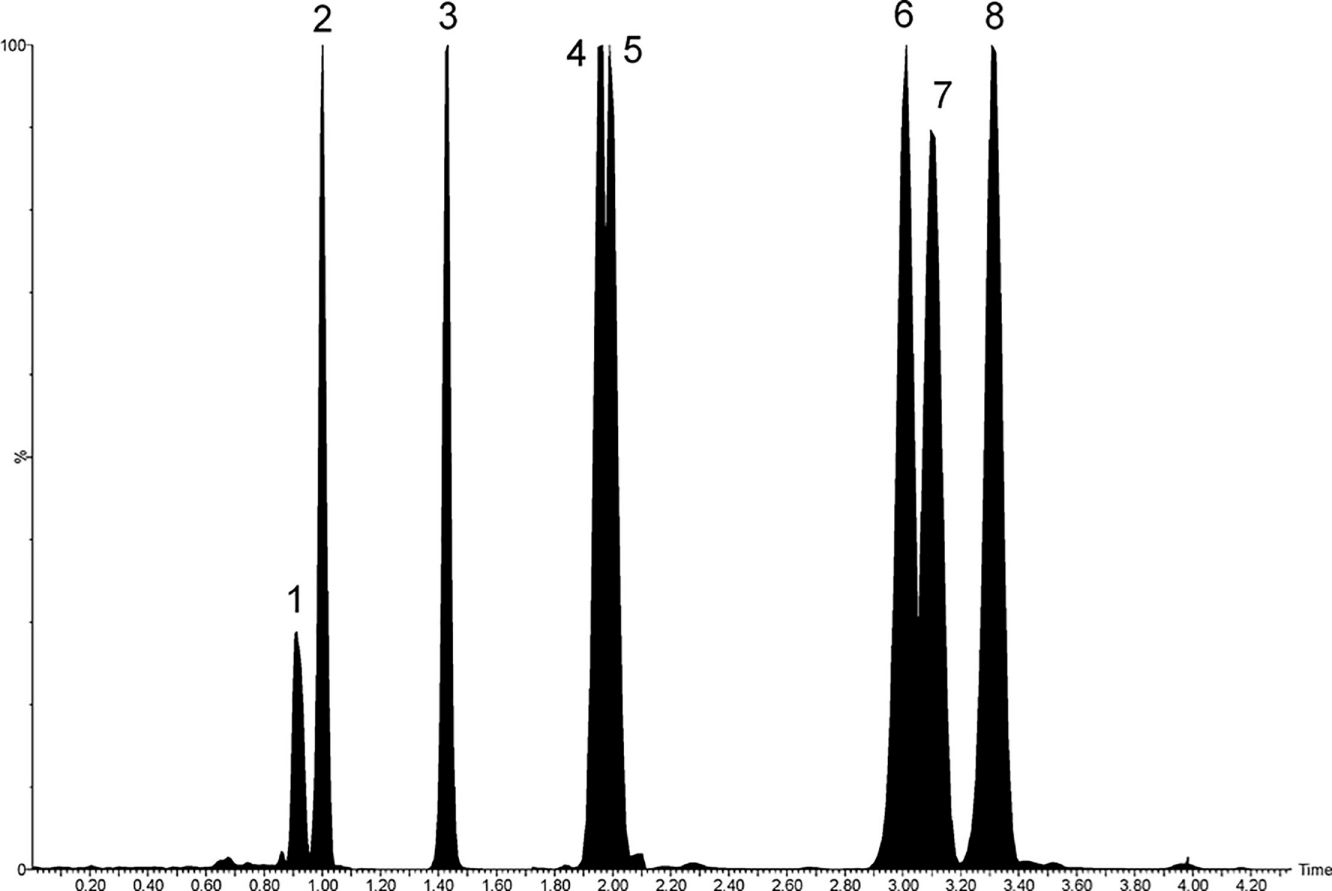
Chromatographic separation of MCPG, HGA, MCPA-carnitine and MCPA-glycine as well as MCPF-carnitine and MCPF-glycine in the serum of an AM horse.

The analysis was conducted according to Sander et al. (2017) [16]. In brief, analysis of MCPG content was performed after butylation on a Xevo UPLC-MS/MS system (Waters, Eschborn, Germany). For chromatographic separation, we used an Acquity UPLC BEH C18 1.7 μm, 2.1x50 mm column (Waters) with an injection volume of 5 μL. The gradient was composed of acetonitrile/water modified by 0.1% formic acid and 0.01% trifluoroacetic acid. MCPG was chromatographically separated into two diastereomers (Fig 2); however, due to the lack of authentic material, it was not possible to assign the peaks to known isomers. Therefore, these products are hereafter referred to as isomers A and B. The analysed transitions [m/z] were 184.0>110.7 for the butyl ester of MCPG and 187.0>113.7 for butylated [^13^C_2_^15^N]-MCPG. The butyl esters were detected in ESI positive mode by multiple reaction monitoring (MRM) mode. A ratio was calculated from the signals that were obtained for both MCPG isomers and the internal [^13^C_2_^15^N]-MCPG standard. The analysed transitions [m/z] for the other butyl esters were MCPF-glycine: 212>80.93, ^13^C_2_^14^N MCPF-glycine: 215.07>80.93, MCPF-carnitine: 298.15>84.98, MCPA-glycine: 226>73.95, ^13^C_2_^14^N MCPA-glycine: 229.1>75.92, and MCPA-carnitine: 312.2>84.98.

Details of the analysis of MCPG and the relevant quality parameters will be published separately.

As shown in Fig 2, MCPG, HGA and the corresponding derivatives in the serum of an affected horse were well separated by chromatography. Chromatographic separation of these compounds was achieved within a run time of 4 min. To also measure a spectrum of acyl carnitines and acyl glycines, the total run time was extended to 14 min.

### Statistical analyses

Statistical analysis was performed with the SAS 9.4 software package (SAS Institute Inc., Cary, NC, USA). All evaluated parameters in serum and urine samples were compared between affected animals and controls using a one sample one sided upper t-test calculating means and standard deviation. Furthermore, to calculate any correlation between the parameters in serum or urine and between the same parameter in serum and urine, a test of normal distribution was performed. Because of the wide ranges in min- and max-values, raw data were logarithmically transformed and tested again for a normal distribution. If log data showed a normal distribution, Pearson correlation coefficients (r) were used to calculate the *P-*value for the t-test to identify significant differences between the samples at *P <* 0.05. Trends are indicated by *P <* 0.1.

## Results

### Quality parameters

Precision data for MCPF-glycine and MCPA-glycine quantification are shown in Table 2. Excellent linearity was found for MCPF-glycine in the range of concentrations measured both in spiked urine and serum, with r^2^ values of 0.9925 and 0.9885 and 0.9784 and 0.9925 for MCPG-glycine (Table 2). The line almost went through the origin, indicating a very low non-specific signal. The limit of quantification was 50 nmol/L (CV < 20%), and the limit of detection, indicated by a signal three times higher than the background, was 5 nmol/L. Recovery of 92–114% of MCPF-glycine and MCPA-glycine from urine and serum spiked with 50 and 500 nmol/L indicated good accuracy of the method. Due to the lack of original compound values obtained for MCPF-carnitine and MCPA-carnitine, the values do not represent an absolute quantification but rather a relative quantification of their concentration in the samples.

**Table 2:** Precision of MCPF-glycine and MCPA-glycine quantification: Coefficients of variation for MCPF-glycine and MCPA-glycine measurements found for 6 concentration levels.

Methylenecyclopropylformylglycine (MCPF-glycine)			Methylenecyclopropylacetylglycine (MCPA-glycine)		
Concentration nmol/L	CV^1^ % serum	CV^1^ % urine	Concentration nmol/L	CV^1^ % serum	CV^1^ % urine
50	18.5	18.7	50	19.8	19.9
100	16.6	16.1	100	17.5	19.6
250	12.7	14.9	250	13.5	14.6
500	6.0	8.6	750	6.9	7.5
1000	4.5	6.8	1250	6.0	8.8
1500	4.0	5.3	2500	3.6	6.8

^1^ coefficients of variation

### HGA in the Acer seeds

HGA concentrations were extremely variable in the *Acer* seeds, ranging from 69–1340 μg/g per seed.

### Serum samples

Serum concentrations of MCPG and HGA and the corresponding metabolites plus the concentrations of a spectrum of acyl conjugates of AM affected horses are shown in Table 3. The concentrations of MCPG in serum samples of all affected horses were extremely variable. In several samples, isomer A was found in trace amounts only. MCPG was always found at a much lower concentration than HGA. The quantitative ratio of the isomers was variable as well. The metabolite MCPF-glycine was found in all serum samples of the affected horses and was detected in concentrations that were different from sample to sample. Because the concentrations for all parameters differed from one another by one or two orders of magnitude, the calculated means were lower than their corresponding standard deviations (e.g., MCPF-glycine and MCPF-carnitine, MCPA-glycine). This was also true for MCPG isomer A and B.

**Table 3:** Concentrations and means ± sd of MCPG and HGA and the corresponding metabolites in nmol/L plus the concentrations of a spectrum of acyl conjugates in μmol/L in the serum samples of AM affected horses in comparison to the controls.

item	AM horse		1	2	3	4	5	6	7	8	9	10	11	12	13	14	Mean^3^±sd
	Units	controls															
**MCPG isomer A**	[nmol/l]	< dl^1^	trace	**1,623**	6.6	trace	36.1	trace	trace	trace	200	8.1	**3.8**	Trace	742	trace	188±458
**MCPG isomer B**	[nmol/l]	< dl^1^	14.7	**228**	6.5	5.2	47.3	6.7	**3.4**	7.6	106	22.4	7.2	7.5	71.5	3.5	38.4±62.6
MCPF-glycine	[nmol/l]	< dl^1^	1,111	652	100	**1,180**	387	97.4	63.8	**52.3**	116	127	153	588	165	123	351±387
MCPF-carnitine	[nmol/l]	< dl^1^	17,832	**990**	3,215	**18,903**	4,938	3,277	1,364	1,649	2,304	10,803	2,371	12,108	2,979	1,060	5,985±6,258
**Hypoglycin A**	[nmol/l]	< dl^1^	3,709	**16,294**	1,427	1,436	10,456	2,439	1,832	2,842	10,753	5,884	**1,184**	1,662	13,050	2,834	5,414±5,056
MCPA-glycine	[nmol/l]	< dl^1^	**4,649**	2,764	241	2,332	1,196	252	**170**	244	681	615	244	876	641	409	1,094±1,296
MCPA-carnitine	[nmol/l]	< dl^1^	**1,025**	171	113	533	379	142	72.5	**70.3**	188	437	93.9	548	510	90.8	312±276
isobutyryl-carnitine	[μmol/l]	0.1–5.3	**36.2**	**1.7**	9.5	29.2	14.2	11.9	7.3	6.9	9.0	25.3	7.5	23.5	10.6	5.8	14.2±10.2
butyryl-carnitine	[μmol/l]	0.2–3.3	37.2	14.3	16.0	32.2	23.6	18.5	16.1	27.3	14.4	28.2	**13.8**	**260**	257	120	62.7±87.1
isovaleryl-carnitine	[μmol/l]	0.2–1.2	11.1	4.8	5.3	6.0	6.8	**4.6**	4.7	4.9	5.0	7.7	5.0	75.7	**166**	18.9	23.3±45.1
valeryl-carnitine	[μmol/l]	trace-dl^2^	**7.3**	**0.5**	0.7	3.6	3.4	1.0	0.7	**0.5**	0.7	4.6	0.7	2.1	4.9	0.9	2.3±2.1
valeryl-glycine	[μmol/l]	trace-0.1	**25.7**	18.2	2.0	14.3	11.4	1.2	**1.0**	3.0	5.0	3.7	2.5	5.4	4.0	3.5	7.2±7.4
hexanoyl-carnitine	[μmol/l]	trace-3.1	13.6	5.8	5.5	14.4	9.1	8.2	7.0	**3.5**	6.8	10.5	6.1	**17.7**	8.1	8.4	8.9±3.9
hexanoyl-glycine	[μmol/l]	<dl^2^-0.4	**35.3**	23.5	2.5	17.8	9.9	2.4	2.7	**1.0**	5.1	4.4	5.6	8.4	7.1	13.6	10.0±9.7
octanoyl-carnitine	[μmol/l]	<dl^2^-1.1	3.7	1.8	1.0	4.1	2.2	2.1	1.5	**0.4**	1.9	2.8	1.2	**4.8**	2.0	2.0	2.2±1.2
decenoyl-carnitine	[μmol/l]	<dl^2^-0.3	3.7	1.7	0.8	3.8	1.5	1.6	1.0	0.4	0.9	2.4	0.9	4.3	1.3	1.6	1.9±1.2

AM-horse: horse affected by atypical myopathy

^1^dl, detection limit = 1 nmol/L

^2^dl = 0.01 μmol/L

^3^calculated as the mean from the horses 1–14; for each parameter the min- and max-values are shown in bold

Neither MCPF- or MCPA-conjugates nor MCPG or HGA were found in the serum samples of healthy control horses. Values for the acyl conjugates for the controls are given in Table 3 and differed significantly from affected horses (*P <* 0.001).

The calculated Pearson coefficients of correlation for the serum samples are shown in Table 4. There was no correlation between MCPG, the precursor to the effective toxin, and MCPF-glycine and MCPF-carnitine. MCPF-glycine was not the major MCPG-metabolite found; rather, MCPF-carnitine was measured in concentrations that exceeded those of MCPF-glycine by one to two powers of ten.

**Table 4:** Pearson correlation coefficients between the detected parameters in serum samples of the AM affected horses.

	MCPG isomer A	MCPG isomer B	MCPF-glycine	MCPF-carnitine	Hypoglycin A	MCPA-glycine	MCPA-carnitine	isobutyryl-carnitine	butyryl-carnitine	isovaleryl-carnitine	valeryl-carnitine	valeryl-glycine	hexanoyl-carnitine	hexanoyl-glycine	octanoyl-carnitine	decenoyl-carnitine
MCPG isomer A	1.0000	0.92 ***<* .*0001***	0.14 0.6397	-0.26 0.3682	0.84 ***0*.*0002***	0.35 0.2218	0.17 0.5666	-0.43 0.1257	-0.09 0.7541	0.10 0.7301	0.03 0.9111	0.38 0.1741	-0.27 0.3512	0.26 0.3626	-0.06 0.8432	-0.17 0.5522
MCPG isomer B	0.92 ***<* .*0001***	1.0000	0.24 0.4022	-0.13 0.6559	0.92 ***<* .*0001***	0.48 0.0800	0.31 0.2759	-0.31 0.2756	-0.08 0.7774	0.11 0.7318	0.16 0.6954	0.49 0.0725	-0.12 0.6792	0.32 0.2716	0.08 0.7797	-0.03 0.9278
MCPF-glycine	0.14 0.6397	0.24 0.4022	1.0000	0.62 0.0177	0.12 0.6933	0.92 ***<* .*0001***	0.76 ***0*.*0016***	0.36 0.2134	0.21 0.4732	0.20 0.4919	0.56 ***0*.*0365***	0.87 ***<* .*0001***	0.69 0.0059	0.87 ***<* .*0001***	0.72 ***0*.*0035***	0.80 ***0*.*0006***
MCPF-carnitine	-0.26 0.3682	-0.13 0.6559	0.62 ***0*.*0177***	1.00000	-0.21 0.4749	0.53 0.0536	0.84 ***0*.*0002***	0.93 ***<* .*0001***	0.24 0.4145	0.21 0.4756	0.80 ***0*.*0005***	0.44 0.1140	0.80 0.0007	0.35 0.2224	0.67 ***0*.*0086***	0.73 ***0*.*0029***
Hypoglycin A	0.84 ***0*.*0002***	0.92 ***<* .*0001***	0.12 0.6933	-0.21 0.4749	1.00000	0.42 0.1357	0.28 0.3323	-0.30 0.3003	0.05 0.8763	0.17 0.5503	0.20 0.5024	0.42 0.1356	-0.12 0.6900	0.28 0.3305	0.07 0.8236	-0.04 0.8971
MCPA-glycine	0.35 0.2218	0.48 0.0800	0.92 ***<* .*001***	0.53 0.0536	0.42 0.1357	1.0000	0.78 ***0*.*0011***	0.27 0.3449	0.15 0.6162	0.15 0.6053	0.58 ***0*.*0285***	0.96 ***<* .*0001***	0.56 0.0389	0.87 ***<* .*0001***	0.63 ***0*.*0166***	0.69 ***0*.*0060***
MCPA-carnitine	0.17 0.5666	0.31 0.2759	0.76 ***0*.*0016***	0.84 ***0*.*0002***	0.28 0.3323	0.78 ***0*.*0011***	1.00000	0.71 **0.0046**	0.44 0.1190	0.48 0.0790	0.91 ***<* .*0001***	0.68 ***0*.*0074***	0.80 ***0*.*0006***	0.63 ***0*.*0164***	0.78 ***0*.*0010***	0.79 ***0*.*0008***
isobutyryl-carnitine	-0.43 0.1257	-0.31 0.2756	0.35 0.2134	0.93 ***<* .*0001***	-0.30 0.3003	0.27 0.3449	0.71 ***0*.*0046***	1.00000	0.29 0.3185	0.24 0.4106	0.79 ***0*.*0009***	0.19 0.5156	0.74 ***0*.*0026***	0.14 0.6236	0.57 ***0*.*0346***	0.59 ***0*.*0278***
butyryl-carnitine	-0.09 0.7541	-0.08 0.7774	0.21 0.4732	0.24 0.4145	0.05 0.8763	0.15 0.6162	0.44 0.1190	0.29 0.3185	1.00000	0.95 ***<* .*0001***	0.47 0.0885	0.11 0.7086	0.49 0.0729	0.25 0.3909	0.39 0.1641	0.42 0.1359
isovaleryl-carnitine	0.10 0.7301	0.10 0.7318	0.20 0.4919	0.21 0.4756	0.17 0.5503	0.15 0.6053	0.48 0.0790	0.24 0.4106	0.95 ***<* .*0001***	1.00000	0.49 0.0729	0.10 0.7353	0.44 0.1107	0.25 0.3909	0.39 0.1686	0.36 0.2042
valeryl-carnitine	0.03 0.9111	0.12 0.6954	0.56 ***0*.*0365***	0.80 ***0*.*0005***	0.20 0.5024	0.58 ***0*.*0285***	0.91 ***<* .*0001***	0.79 ***0*.*0009***	0.47 0.0885	0.49 0.0729	1.00000	0.49 0.0726	0.75 ***0*.*0020***	0.49 0.0720	0.68 ***0*.*0075***	0.69 ***0*.*0058***
valeryl-glycine	0.39 0.1741	0.49 0.0725	0.87 ***<* .*001***	0.44 0.1140	0.42 0.1356	0.96 ***<* .*0001***	0.68 0.0074	0.19 0.5156	0.11 0.7086	0.09 0.7353	0.49 0.0726	1.0000	0.41 0.1419	0.83 ***0*.*0002***	0.46 0.0968	0.54 ***0*.*0440***
hexanoyl-carnitine	-0.27 0.3512	-0.12 0.6792	0.69 ***0*.*0059***	0.80 ***0*.*0007***	-0.12 0.6900	0.56 ***0*.*0389***	0.80 ***0*.*0006***	0.74 ***0*.*0026***	0.49 0.0729	0.45 0.1107	0.75 ***0*.*0020***	0.41 0.1419	1.00000	0.61 ***0*.*0219***	0.96 ***<* .*0001***	0.95 ***<* .*0001***
hexanoyl-glycine	0.26 0.3626	0.32 0.2716	0.87 ***<* .*001***	0.35 0.2224	0.28 0.3305	0.87 ***<* .*0001***	0.63 ***0*.*0164***	0.14 0.6236	0.25 0.3909	0.25 0.3909	0.49 0.0720	0.83 ***0*.*0002***	0.60 ***0*.*0219***	1.00000	0.71 ***0*.*0049***	0.73 ***0*.*0031***
octanoyl-carnitine	-0.06 0.8432	0.08 0.7797	0.72 ***0*.*0035***	0.67 ***0*.*0086***	0.07 0.8236	0.63 ***0*.*0166***	0.78 ***0*.*0010***	0.57 ***0*.*0346***	0.39 0.1641	0.39 0.1686	0.68 ***0*.*0075***	0.46 0.0968	0.95 ***<* .*0001***	0.70 ***0*.*0049***	1.0000	0.95 ***<* .*0001***
decenoyl-carnitine	-0.17 0.5522	-0.03 0.9278	0.80 ***0*.*0006***	0.73 ***0*.*0029***	-0.04 0.8971	0.69 ***0*.*0060***	0.79 ***0*.*0008***	0.59 ***0*.*0278***	0.42 0.1359	0.36 0.2042	0.69 ***0*.*0058***	0.54 ***0*.*0440***	0.95 ***<* .*0001***	0.73 ***0*.*0031***	0.95 ***<* .*0001***	1.00000

significant differences in t-tests are shown as bold/italic *P-*values (*P <* 0.05) in every column below the correlation coefficient; grey-coloured columns show the correlations between the acyl glycines and acyl carnitines

Unlike MCPG and its metabolites, the parent substance HGA was present in the serum at a much higher concentration than the respective glycine and carnitine conjugates in the group of affected horses. There was a strong relationship between the abundance of HGA and MCPG (r = 0.84–0.92; MCPG A isomer and MCPG isomer B), and accordingly, levels of MCPF-carnitine exceeded those of MCPA-carnitine by up to more than two orders of magnitude, and both were strongly correlated (r = 0.84). The detected concentrations of the medium chain acyl conjugates were extremely high in comparison to the control horses (*P <* 0.001), and in some cases, there was a strong correlation to the toxic metabolites. For example, there was a strong correlation between the abundance of MCPF-glycine and valeryl-glycine (r = 0.87) and hexanoyl-glycine (r = 0.87), or between MCPA-glycine and valeryl-glycine (r = 0.96) and hexanoyl-glycine (r = 0.87), and between MCPA-carnitine and valeryl-carnitine (r = 0.92). This correlation was also found for the abundance of MCPF-carnitine and isobutyryl-carnitine (r = 0.93).

### Urine samples

MCPG was excreted in the urine of the AM affected horses only in very small amounts, and sometimes only traces of the compounds were detected (Table 5). Concentrations were about two orders of magnitude lower for MCPG than for HGA; however, MCPF-glycine and MCPF-carnitine were found in all urine samples of affected horses in high concentrations in addition to the respective MCPA conjugates. As described for serum samples, it can be noted that the calculated mean for HGA and MCPA-glycine was lower than the corresponding standard deviation, which can be explained by the high variation among the detected concentrations in the AM horses. This observation was also true for MCPG isomer A and B; the detected concentrations, however, were clearly lower.

**Table 5:** Concentrations and means ± sd of MCPG and HGA and the corresponding metabolites in nmol/mmol creatine plus concentrations of a spectrum of acyl conjugates in μmol/mmol creatinine in urine samples of AM affected horses in comparison to the controls.

item	AM horse		1	2	3	4	5	6	7	8	9	10	11	12	13	14	mean^3^±sd
	Units	controls															
**MCPG isomer A**	nmol/mmol crea	< dl^1^	1.8	10.1	**1.2**	1.3	2.5	5.1	13.0	trace	**1.2**	1.6	trace	3.1	**25.5**	0	4.8±7.1
**MCPG isomer B**	nmol/mmol crea	< dl^1^	trace	trace	trace	3.3	**2.7**	trace	7.1	trace	2.5	11.8	trace	7.7	**51.3**	trace	6.5±13.4
MCPF-glycine	nmol/mmol crea	< dl^1^	4,673	**16,058**	6,394	7,509	7,624	3,518	**1,771**	9,399	6,640	1,832	5,790	8,724	2,757	3,846	6,181±3,758
MCPF-carnitine	nmol/mmol crea	< dl^1^	17,478	**5,380**	10,297	14,632	7,271	14,397	6,668	8,395	8,672	**29,378**	11,906	17,824	17,456	23,887	13,831±6,930
**Hypoglycin A**	nmol/mmol crea	< dl^1^	**3,405**	1,344	244	553	**63.8**	218	177	722	630	1,142	241	838	518	527	759±847
MCPA-glycine	nmol/mmol crea	< dl^1^	81,300	**192,704**	28,122	25,402	60,505	16,849	**12,922**	17,889	68,766	25,174	21,005	34,548	27,595	22,209	45,356±47,283
MCPA-carnitine	nmol/mmol crea	< dl^1^	2,701	1,097	405	687	1,538	1,219	537	**133**	743	**2,754**	473	798	2,392	836	1,165±864
isobutyryl-carnitine	μmol/mmol crea	0.8–1.3	53.0	**14.0**	52.5	50.3	26.2	97.7	54.0	27.9	73.2	**125**	54.0	69.6	38.3	59.4	56.8±28.9
butyryl-carnitine	μmol/mmol crea	trace-0.3	**672**	207	145	223	542	606	462	**131**	181	499	171	272	313	408	345±184
isovaleryl-carnitine	μmol/mmol crea	trace-0.6	**240**	84.9	72.1	74.5	214.0	167	109	**56.1**	81.4	200	80.9	101	110	124	123±58.9
valeryl-carnitine	μmol/mmol crea	trace<dl^2^	19.4	8.7	3.7	4.9	8.6	8.0	3.8	**1.3**	5.7	22.1	4.5	4.9	**24.8**	8.9	9.2±7.4
valeryl-glycine	μmol/mmol crea	0.8–5.3	365	**1079**	263	201	574	**64.1**	86.7	106	410	145	192	307	74.0	135	286±272
hexanoyl-carnitine	μmol/mmol crea	trace-0.8	168	80.8	47.9	76.6	118	116	52.9	**14.0**	69.6	**173**	72.5	81.3	82.2	90.1	88.7±43.3
hexanoyl-glycine	μmol/mmol crea	0.4–0.8	964	**1158**	294	343	578	190	282	**184**	389	245	376	502	456	438	457±282
octanoyl-carnitine	μmol/mmol crea	trace-0.3	21.0	22.3	10.2	16.5	**26.3**	17.6	9.8	**3.0**	14.9	23.3	15.6	15.7	16.3	16.4	16.4±6.01
decenoyl-carnitine	μmol/mmol crea	trace<dl^2^	10.5	**20.7**	8.1	10.4	19.2	7.3	4.6	**1.9**	7.8	12.7	12.5	13.5	11.4	14.4	11.1±5.12

AM-horse: Horse affected by atypical myopathy

^1^dl, detection limit = 1 nmol/mmol creatinine

^2^dl = 0.01 μmol/mmol creatinine

^3^calculated as the mean from the horses 1–14; for each parameter the min- and max-values are shown in bold

In urine, the levels of MCPF-carnitine were significantly higher than those of MCPF-glycine. The urine results from all affected horses indicated that acyl glycines and acyl carnitines were present in extremely elevated concentrations. Neither HGA, MCPG, MCPF- nor MCPA-conjugates were detected in the urine of healthy control horses. Control horses excreted acyl glycines and acyl carnitines at concentrations (Table 5), which were significantly different from those measured in affected horses (*P <* 0.001).

Table 6 shows a strong correlation in urine samples between MCPA-glycine and valeryl-glycine (r = 0.89) and hexanoyl-glycine (r = 0.85), as well as between MCPA-carnitine and isovaleryl-carnitine (r = 0.81), valeryl-carnitine (r = 0.97), hexanoyl-carnitine (r = 0.91) and octanoyl-carnitine (r = 0.85). Regarding the specific MCPF-conjugates, there are only low correlations in the urine samples. There are some correlations between the acyl glycines and acyl carnitines, which can be found in Table 6.

**Table 6:** Pearson correlation coefficients between the detected parameters in urine samples of the AM affected horses.

	MCPG isomer A	MCPG isomer B	MCPF-glycine	MCPF-carnitine	Hypoglycin A	MCPA-glycine	MCPA-carnitine	isobutyryl-carnitine	butyryl-carnitine	isovaleryl-carnitine	valeryl-carnitine	valeryl-glycine	hexanoyl-carnitine	hexanoyl-glycine	octanoyl-carnitine	decenoyl-carnitine
MCPG isomer A	1.00000	0.43 0.1273	-0.13 0.6470	-0.30 0.2947	-0.06 0.8291	0.23 0.4311	0.41 0.1423	-0.14 0.6384	0.22 0.4581	0.18 0.5459	0.32 0.2691	0.06 0.8353	0.24 0.4144	0.20 0.4961	0.28825 0.3176	0.13 0.6519
MCPG isomer B	0.43 0.1273	1.00000	-0.46 0.0950	0.25 0.3883	-0.18 0.5473	-0.28 0.3284	0.29 0.3177	0.23 0.4359	0.12 0.6750	0.04 0.8823	0.29 0.3181	-0.34 0.2346	0.10 0.7348	-0.21 0.4705	0.11 0.6980	0.08 0.7832
MCPF-glycine	-0.13 0.6470	-0.46 0.0950	1.00000	-0.49 0.0787	0.12 0.6722	0.59 ***0*.*0267***	-0.38 0.1793	-0.64 ***0*.*0136***	-0.57 ***0*.*0302***	-0.46 0.0960	-0.43 0.1295	0.70 ***0*.*0049***	-0.33 0.2503	0.42 0.1389	-0.11 0.7050	0.14 0.6353
MCPF-carnitine	-0.30 0.2947	0.25 0.3883	-0.49 0.0787	1.00000	0.36 0.2030	-0.32 0.2583	0.45 0.1052	0.69 ***0*.*0068***	0.37 0.1955	0.39 0.1639	0.52 0.0549	-0.41 0.1458	0.48 0.0823	-0.16 0.5819	0.28 0.3381	0.22 0.4589
Hypoglycin A	-0.06 0.8291	-0.18 0.5473	0.12 0.6722	0.36 0.2030	1.00000	0.41 0.1479	0.25 0.3962	0.04 0.9015	-0.02 0.9255	0.03 0.9244	0.31 0.2798	0.20 0.4895	0.15 0.6185	0.38 0.1802	0.04 0.9028	0.03 0.9231
MCPA-glycine	0.23 0.4311	-0.28 0.3284	0.59 0.0267	-0.32 0.2583	0.41 0.1479	1.00000	0.38 0.1823	-0.52 0.0588	-0.03 0.9317	0.15 0.6004	0.33 0.2434	0.89 ***<* .*0001***	0.32 0.2610	0.85 ***0*.*0001***	0.48 0.0836	0.55 ***0*.*0429***
MCPA-carnitine	0.41 0.1423	0.29 0.3177	-0.38 0.1793	0.45 0.1052	0.25 0.3962	0.38 0.1823	1.00000	0.22 0.4581	0.73 ***0*.*0027***	0.81 ***0*.*0004***	0.97 < **.*0001***	0.13506 0.6453	0.91 ***<* .*0001***	0.46 0.0942	0.84 ***0*.*0001***	0.67 ***0*.*0090***
isobutyryl-carnitine	-0.14 0.6384	0.23 0.4359	-0.64 ***0*.*0136***	0.69 ***0*.*0068***	0.04 0.9015	-0.52 0.0588	0.22 0.4581	1.00000	0.33 0.2514	0.31 0.2868	0.20 0.4904	-0.49 0.0748	0.37 0.1918	-0.52 0.0553	0.14 0.6200	-0.09 0.7623
butyryl-carnitine	0.22 0.4581	0.12 0.6750	-0.58 0.0302	0.37 0.1955	-0.03 0.9255	-0.03 0.9317	0.73 ***0*.*0027***	0.33 0.2514	1.00000	0.94 ***<* .*0001***	0.65 ***0*.*0121***	-0.18 0.5430	0.73 ***0*.*0027***	0.15 0.6113	0.56 ***0*.*0356***	0.30 0.3034
isovaleryl-carnitine	0.18 0.5459	0.04 0.8823	-0.46 0.0960	0.39 0.1639	0.03 0.9244	0.15 0.6004	0.81 ***0*.*0004***	0.31 0.2868	0.94 ***<* .*0001***	1.00000	0.73 ***0*.*0028***	0.02 0.9437	0.82 ***0*.*0004***	0.25 0.3887	0.66 ***0*.*0103***	0.42 0.1306
valeryl-carnitine	0.32 0.2691	0.29 0.3181	-0.43 0.1295	0.52 0.0549	0.31 0.2798	0.33 0.2434	0.97 ***<* .*0001***	0.20 0.4904	0.65 ***0*.*0121***	0.73 ***0*.*0028***	1.00000	0.05 0.8532	0.85 ***0*.*0001***	0.44 0.1146	0.78 ***0*.*0010***	0.64 ***0*.*0139***
valeryl-glycine	0.06 0.8353	-0.34 0.2346	0.70 ***0*.*0049***	-0.41 0.1458	0.20 0.4895	0.89 ***<* .*0001***	0.14 0.6453	-0.49 0.0748	-0.18 0.5430	0.02 0.9437	0.05 0.8532	1.00000	0.21 0.4725	0.76 ***0*.*0017***	0.41 0.1510	0.55 ***0*.*0421***
hexanoyl-carnitine	0.24 0.4144	0.10 0.7348	-0.33 0.2503	0.48 0.0823	0.15 0.6185	0.32 0.2610	0.91 ***<* .*0001***	0.37 0.1918	0.73 ***0*.*0027***	0.82 ***0*.*0004***	0.85 ***0*.*0001***	0.21 0.47	1.00000	0.43 0.1274	0.94 ***<* .*0001***	0.76 ***0*.*0016***
hexanoyl-glycine	0.20 0.4961	-0.21 0.4705	0.42 0.1389	-0.16 0.5819	0.38 0.1802	0.85 ***0*.*0001***	0.46 0.0942	-0.52 0.0553	0.15 0.6113	0.25 0.3887	0.44 0.1146	0.76 ***0*.*0017***	0.43 0.1274	1.00000	0.56 ***0*.*0357***	0.68 ***0*.*0075***
octanoyl-carnitine	0.29 0.3176	0.11 0.6980	-0.11 0.7050	0.28 0.3381	0.04 0.9028	0.48 0.0836	0.85 ***0*.*0001***	0.15 0.6200	0.56 ***0*.*0356***	0.66 ***0*.*0103***	0.78 ***0*.*0010***	0.40 0.1510	0.94 ***<* .*0001***	0.56 ***0*.*0357***	1.00000	0.91 ***<* .*0001***
decenoyl-carnitine	0.13 0.6519	0.08 0.7832	0.14 0.6353	0.22 0.4589	0.03 0.9231	0.55 ***0*.*0429***	0.67 ***0*.*0090***	-0.09 0.7623	0.30 0.3034	0.42 0.1306	0.64 ***0*.*0139***	0.55 ***0*.*0421***	0.76 ***0*.*0016***	0.68 ***0*.*0075***	0.91 ***<* .*0001***	1.00000

significant differences in t-tests are shown as bold/italic *P-*values (*P <* 0.05) in every column below the correlation coefficient; grey-coloured columns show the correlations between the acyl glycines and acyl carnitine

A comparison of detected acyl conjugates concentrations between serum and urine samples (Table 7) showed a high correlation for valeryl-carnitine (r = 0.71), valeryl-glycine (r = 0.73), hexanoyl-carnitine (r = 0.70), hexanoyl-glycine (r = 0.87) and octanoyl-carnitine (r = 0.78), which were all in the range of the correlation calculated for MCPA-carnitine (r = 0.73) and MCPA-glycine (r = 0.78). In addition to the fact that there are some correlations between several acyl carnitines in serum (e.g., valeryl-carnitine, hexanoyl-carnitine, octanoyl-carnitine) and the abundance of MCPA-carnitine in urine, as well as for some acyl glycines in serum (valeryl-glycine, hexanoyl-glycine) and MCPA-glycine in urine, there was also a relationship between MCPF-carnitine in urine and isobutyryl-carnitine and butyryl- and valeryl-carnitine in serum samples. The latter observation was similar to the description in the serum samples.

**Table 7:** Pearson correlation coefficients between the detected parameters in serum and urine samples of the AM affected horses.

Serum ►	MCPG isomer A	MCPG isomer B	MCPF-glycine	MCPF-carnitine	Hypoglycin A	MCPA-glycine	MCPA-carnitine	isobutyryl-carnitine	butyryl-carnitine	isovaleryl-carnitine	valeryl-carnitine	valeryl-glycine	hexanoyl-carnitine	hexanoyl-glycine	octanoyl-carnitine	decenoyl-carnitine
Urine ▼																
MCPG isomer A	0.36 0.2126	0.44 0.1144	0.21 0.4728	0.17 0.5688	0.34 0.2285	0.20 0.4858	0.37 0.1950	0.05 0.8624	-0.04 0.8970	0.18 0.5340	0.26 0.3719	0.04 0.8855	0.18 0.5367	0.05 0.8751	0.29 0.3182	0.19 0.5064
MCPG isomer B	0.16 0.5827	0.14 0.6269	-0.12 0.6714	0.15 0.5975	0.24 0.4144	-0.13 0.6649	0.29 0.3146	0.24 0.4127	0.51 0.0626	0.59 ***0*.*0276***	0.38 0.1792	-0.21 0.4746	0.30 0.3042	-0.14 0.6311	0.29 0.3209	0.14 0.6205
MCPF-glycine	0.28 0.3297	0.32 0.2644	0.44 0.1144	-0.06 0.8431	0.11 0.7129	0.40 0.1528	-0.00 0.9996	-0.3 0.2765	-0.13 0.6562	-0.16 0.5755	-0.30 0.2995	0.54 0.0459	-0.16 0.5909	0.25 0.3889	-0.13 0.6569	-0.04 0.9001
MCPF-carnitine	-0.31 0.2882	-0.32 0.2581	0.09 0.7539	0.51 0.0632	-0.21 0.4646	0.06 0.8457	0.43 0.1214	0.60 ***0*.*0230***	0.59 ***0*.*0261***	0.50 0.0676	0.58 ***0*.*0299***	-0.02 0.9344	0.57 ***0*.*0346***	0.18 0.5489	0.47 0.0936	0.51 0.0604
Hypoglycin A	0.09 0.7610	0.21 0.4671	0.39 0.1628	0.30 0.3039	0.17 0.5670	0.56 ***0*.*0394***	0.42 0.1319	0.15 0.6051	0.25 0.3939	0.22 0.4591	0.26 0.3651	0.50 0.0667	0.25 0.3930	0.42 0.1312	0.26 0.3721	0.40 0.1562
MCPA-glycine	0.65 ***0*.*0118***	0.79 ***0*.*0008***	0.62 ***0*.*0184***	0.05 0.8537	0.66 ***0*.*0103***	0.78 ***0*.*0011***	0.41 0.1438	-0.21 0.4674	-0.12 0.6752	-0.03 0.9236	0.13 0.6651	0.80 ***0*.*0007***	0.11 0.7060	0.66 ***0*.*0100***	0.28 0.3311	0.27 0.3528
MCPA-carnitine	0.33 0.2539	0.43 0.1288	0.45 0.1102	0.43 0.1289	0.51 0.0623	0.54 0.0449	0.73 ***0*.*0031***	0.37 0.1929	0.28 0.3268	0.39 0.1725	0.75 ***0*.*0019***	0.39 0.1676	0.61 ***0*.*0203***	0.59 ***0*.*0273***	0.72 ***0*.*0037***	0.64 ***0*.*0128***
isobutyryl-carnitine	-0.49 0.0734	-0.45 0.1090	-0.26 0.3691	0.39 0.1596	-0.41 0.1481	-0.32 0.2618	0.12 0.6914	0.59 ***0*.*0264***	0.10 0.7334	0.06 0.8457	0.23 0.4345	-0.45 0.1041	0.40 0.1483	-0.25 0.3971	0.34 0.2362	0.25 0.3798
butyryl-carnitine	-0.24 0.4166	-0.08 0.7906	0.18 0.5386	0.30 0.2908	0.16 0.5877	0.22 0.4517	0.41 0.1499	0.41 0.1483	0.17 0.5565	0.15 0.6165	0.57 0.0332	0.06 0.8334	0.54 ***0*.*0476***	0.30 0.2911	0.55 ***0*.*0425***	0.52 0.0545
isovaleryl-carnitine	-0.09 0.7685	0.09 0.7594	0.25 0.3927	0.40 0.1565	0.27 0.3524	0.32 0.2646	0.52 0.0571	0.46 0.0968	0.11 0.6984	0.13 0.6700	0.65 ***0*.*0116***	0.21 0.4814	0.52 0.0556	0.36 0.2116	0.54 ***0*.*0486***	0.51 0.0623
valeryl-carnitine	0.39 0.1647	0.43 0.1254	0.35 0.2160	0.32 0.2589	0.53 0.0522	0.48 0.0832	0.66 ***0*.*0109***	0.28 0.3251	0.34 0.2415	0.44 0.1121	0.71 ***0*.*0042***	0.35 0.2242	0.50 0.0691	0.56 ***0*.*0361***	0.61 ***0*.*0203***	0.55 ***0*.*0439***
valeryl-glycine	0.44 0.1119	0.57 ***0*.*0326***	0.61 ***0*.*0203***	0.10 0.7428	0.39 0.1730	0.66 ***0*.*0105***	0.28 0.3294	-0.17 0.5571	-0.24 0.4001	-0.19 0.5055	-0.00 0.9928	0.73 ***0*.*0028***	0.12 0.6878	0.58 ***0*.*0288***	0.24 0.4172	0.23 0.4244
hexanoyl-carnitine	0.12 0.6836	0.22 0.4446	0.49 0.0737	0.48 0.0856	0.25 0.3812	0.49 0.0742	0.63 ***0*.*0150***	0.41 0.1414	0.13 0.6572	0.19 0.5212	0.64 ***0*.*0137***	0.34 0.2286	0.70 ***0*.*0053***	0.63 ***0*.*0164***	0.81 ***0*.*0005***	0.73 ***0*.*0031***
hexanoyl-glycine	0.44 0.1115	0.54 ***0*.*0459***	0.74 ***0*.*0025***	0.08 0.7746	0.46 0.0972	0.78 ***0*.*0009***	0.47 0.0896	-0.14 0.6276	0.16 0.5809	0.24 0.4006	0.27 0.3542	0.79 ***0*.*0009***	0.32 0.2697	0.87 ***<* .*0001***	0.45 0.1107	0.45 0.1043
octanoyl-carnitine	0.32 0.2615	0.39 0.1709	0.58 ***0*.*0313***	0.35 0.2201	0.36 0.2033	0.55 ***0*.*0406***	0.58 ***0*.*0292***	0.22 0.4576	0.08 0.7772	0.17 0.5655	0.52 0.0557	0.44 0.1167	0.63 0.0165	0.71 ***0*.*0044***	0.78 ***0*.*0009***	0.68 ***0*.*0076***
decenoyl-Carnitine	0.40 0.1582	0.41 0.1507	0.61 ***0*.*0203***	0.21 0.4686	0.34 0.2343	0.54 ***0*.*0455***	0.48 0.0824	0.02 0.9468	0.21 0.4683	0.28 0.3322	0.38 0.1765	0.50 0.0652	0.52 0.0581	0.75 ***0*.*0018***	0.66 ***0*.*0101***	0.60 ***0*.*0247***

significant differences in t-tests are shown as bold/italic *P-*values (*P <* 0.05) in every column below the correlation coefficient; light grey-coloured boxes show the correlations between the acyl glycines and acyl carnitines; dark-grey coloured boxes show the correlation between the abundance of the individual parameter in serum in comparison to urine

## Discussion

So far, only HGA has been reported to cause AM in horses, although it has been known for a long time that fruits from *Sapindaceae* not only contain HGA but also the structural analogue MCPG [12]. Previous studies detailed the relationship between HGA content in seeds of *Acer pseudoplatanus* ingested by mainly younger horses (< 3 years) on pastures and the detectable concentrations of HGA and the toxic metabolites (MCPA-glycine and MCPA-carnitine) in serum and urine after developing AM [3, 4, 31–33]. The repeated observation of the prevalence in young horses has not yet been fully explained, but may be due to the fact that horses until the age of three years spend more time on pasture over the whole year and have a higher susceptibility because of the higher energy need during the growth period [31].

In the present study, it has been observed that besides HGA, MCPG can also play a role in the development of AM in horses. The MCPG concentrations in serum and urine were quite low in comparison to HGA, but in contrast, the concentrations of the corresponding metabolites were extremely high. A possible explanation for the low MCPG content in body fluids could be fast and complete metabolism to its metabolites after absorption in the digestive tract. The serum and urine samples from affected horses of the present study were from the latest available time point during the disease before the horses were euthanized or deceased. It is most likely that at this time point the majority of available MCPG was metabolized.

The relative importance of MCPG or HGA has not yet been clarified. The toxic effects could be species-specific and organ-specific. Additionally, nothing is known about the distribution of toxins in the various organs in different species. After ingestion, MCPG and HGA are metabolized through several steps to produce the acidic compounds MCPF and MCPA, which are then enzymatically transformed to the CoA-thioesters MCPF-CoA and MCPA-CoA [25, 34–35]. The mechanism of enzyme inhibition by the CoA-thioesters is probably best explained by irreversible or at least firm binding of the compounds to the active sites of enzymes responsible for the of β-oxidation of fatty acids [36]. While MCPA-CoA is a potent inhibitor of acyl-CoA dehydrogenases and interrupts the first step of the β-oxidation cycle, MCPF-CoA is able to inhibit the first step of β-oxidation only to a certain extent. Enzymes affected by MCPA-CoA are isovaleryl-CoA dehydrogenase and 2-methyl-branched chain acyl-CoA dehydrogenase. The predominant effect of MCPF-CoA is to block enoyl-CoA hydratases (ECH) that are responsible for the reversible hydration of 2-trans-enoyl-CoA thioesters to the corresponding hydroxyacyl-compounds. This second step is performed by ECH 1, which is localized in the mitochondria, and ECH 2, which is bound to peroxisomes. ECH 1 is active as a mono-functional mitochondrial enzyme, but it is also part of the so-called mitochondrial trifunctional protein that catalyse β-oxidation of long chain acyl compounds in three consecutive steps. Both the mono-functional and tri-functional proteins are inhibited by MCPF-CoA. ECH 2 does not exist as a mono-functional enzyme but is integrated into a multi-functional peroxisomal enzyme complex that is an essential factor of peroxisomal β-oxidation, as it acts on a wide variety of acyl compounds. MCPF-CoA occurs in nature in two isomeric forms; while (R)-MCPF-CoA is more effective in inhibiting ECH 2 than (S)-MCPF-CoA, the effects of both stereoisomers on ECH 1 are similar [13, 24]. In the small group of affected animals tested, we observed a statistically strong correlation between MCPF-glycine and the acyl conjugates valeryl-glycine and hexanoyl-glycine in serum samples; however, this correlation was also observed for MCPA-glycine. An isolated effect of the abundance of MCPF-carnitine in serum was observed by a strong correlation to isobutyryl-carnitine. Therefore, we hypothesize that this acyl carnitine mainly accumulates due to the presence of MCPF-CoA. In the urine samples, we observed a strong correlation for MCPA-conjugates and several acyl conjugates. We speculate that the simultaneous effect of the toxins derived from the two amino acids HGA and MCPG reinforces the inhibition of β-oxidation. However, the exact biochemical process of the inhibition of energy production in AM remains to be elucidated. An increasing number of affected animals may strengthen this observation.

There are still more unresolved questions about the genesis of AM. The most disturbing phenomenon is that on a pasture contaminated with Acer seeds, only some animals become seriously ill, while others remain clinically unremarkable. In accordance with the present study it has been observed that especially young individuals are affected [3, 4]. This phenomenon appears to be a fundamental principle of poisoning by plants containing HGA and MCPG. In human studies, it was also observed that often only single individuals, mostly younger ones, are diseased while others are not affected. This was reported as early as 1917 by Scott [6] for ackee meals and later for lychee consumption [37–39]. Fruit or fruit products in such events were equally available to all and often were consumed together. Since interrupting energy production is a key mechanism of toxicity, biochemical stress, triggered by physical exertion and hunger or fever, could determine if a horse that has eaten maple seeds becomes clinically ill. Additionally, accompanying substances, which are ingested together with HGA and MCPG, might play a role.

With regard to AM, not only the factors mentioned must be discussed here. It is also important to remember that the content of HGA, as shown here, and MCPG may be extremely variable in the individual maple seeds. In comparison to other *Sapindaceae* (lychee fruits, MCPG 45–220 μg/g; HGA 12–152 μg/g) the observed levels reported here are clearly higher. The degree of maturity and the amount of seeds ingested by the affected horses are most likely essential for poisoning and the development of AM symptoms. Possibly only a few seeds containing the highest HGA concentrations are sufficient to severely poison a horse. Thus, the different incidence of AM may be due to individual sensitivity as well as to random differences in the uptake of HGA, MCPG and accompanying substances.

As shown here, measurement of MCPG can effectively be included into a method developed for the diagnosis of *Acer* seed poisoning. MCPG, HGA and their specific metabolites were detected in serum as well as in urine samples. For practical application, it has to be considered that concentrations of MCPG were low in comparison to HGA, but the method is sensitive enough to even detect traces of the toxin. For example, as it has been previously demonstrated, HGA, but not the toxic metabolites, can be found in serum samples of obviously healthy co-grazers staying on the same pasture as affected ones [32]. An explanation for this observation is still missing, but it can be speculated that the risk for developing an AM is much higher for those horses. Therefore this group of horses cannot act as controls in future studies, but need much more attention. A full biochemical diagnosis of AM requires not only measurement of HGA and MCPG and their metabolites but also analysis of a spectrum of acyl conjugates. The method presented here allows for a full analysis with an instrumental run time of only 14 min.

## Conclusions

In this study, it has been demonstrated that in addition to HGA, MCPG is involved in *Acer* seed poisoning of horses. This finding has to be taken into account for the description of the exact pathomechanism of AM. A deeper insight into the pathogenesis is important from the point of view of applied veterinary medicine because only a precise understanding of pathogenetic mechanisms will lead to the development of a specific therapy, which is still missing.

Serum and urine sampling were suitable for quantitation of MCPG and the corresponding MCPF-metabolites. The high sensitivity of the method makes it easy to quantify low concentrations of the relevant compounds and typical metabolites that may be observed in subclinical or symptom-free cases.

In our opinion, the demonstration of the involvement of MCPG in AM in horses is one step only towards a complete elucidation of the pathogenesis of the disease. Further investigations are required. Those studies should address MCPG plus HGA in the body fluids of affected horses, as well as of symptom-free, co-grazing horses, to estimate the risk that may be associated with the consumption of maple constituents.

## References

[1] Westermann CM, Dorland L, Votion DM, de Sain-van der Velden MG, Wijnberg ID, Wanders RJ et al (2008): Acquired multiple Acyl-CoA dehydrogenase deficiency in 10 horses with atypical myopathy. *Neuromuscul Disord*. 18: 355–64 10.1016/j.nmd.2008.02.007 18406615

[2] Sponseller BT, Valberg SJ, Schultz NE, Bedford H, Wong DM, Kersh K et al (2012): Equine multiple acyl-CoA dehydrogenase deficiency (MADD) associated with seasonal pasture myopathy in the midwestern United States. *J Vet Intern Med*. 26:1012–18 10.1111/j.1939-1676.2012.00957.x 22708588

[3] Valberg SJ, Sponseller BT, Hegeman AD, Earing J, Bender JB, Martinson KL et al (2013): Seasonal pasture myopathy/atypical myopathy in North America associated with ingestion of hypoglycin A within seeds of the box elder tree. *Equine Vet J*. 45: 419–426 10.1111/j.2042-3306.2012.00684.x 23167695

[4] Votion DM, van Galen G, Sweetman L, Boemer F, de Tullio P, Dopagne C http://www.ncbi.nlm.nih.gov/pubmed/?term=Dopagne%20C%5BAuthor%5D&cauthor=true&cauthor_uid=23773055 et al (2014): Identification of methylenecyclopropyl acetic acid in serum of European horses with atypical myopathy. *Equine Vet J*. 46:146–149 10.1111/evj.12117 23773055

[5] Sander J, Cavalleri JM, Terhardt M, Bochnia M, Zeyner A, Zuraw A et al (2016a): Rapid diagnosis of hypoglycin A intoxication in atypical myopathy of horses. *J Vet Diagn Invest*. 28: 98–104 26965229 10.1177/1040638715624736

[6] Scott H, **(**1916) On the vomiting sickness of Jamaica. *Annals of tropical medicine and parasitology* 10: 1–78

[7] Hassall CH, Reyle K (1955): The toxicity of the ackee (*Blighia sapida*) and it relationship to the vomiting sickness of Jamaica; a review. *West Indian Med J*. 4: 83–90 13257078

[8] Joskow R, Belson M, Vesper H, Backer L, Rubin C **(**2006): Ackee fruit poisoning: an outbreak investigation in Haiti 2000–2001, and review of the literature. *Clin Toxicol (Phila)*. 44: 267–73 16749544 10.1080/15563650600584410

[9] Barceloux DG (2009): Ackee fruit and Jamaican vomiting sickness (Blighia sapida Köenig). *Dis Mon*. 55: 318–26 10.1016/j.disamonth.2009.03.002 19446675

[10] Hassall CH, Reyle K (1954): Hypoglycin A, B: biologically active polypeptides from Blighia sapida. *Nature* 173 (4399): 356–357 10.1038/173356b0 13144762

[11] Gray DO, Fowden L (1962): alpha-(Methylenecyclopropyl)glycine from Litchi seeds. *Biochem J*. 82: 385–389 13901296 10.1042/bj0820385 PMC1243468

[12] Fowden L, Pratt HM (1973): Cyclopropyl amino acids of the genus Acer: Distribution and biosynthesis. *Phytochemistry* 12: 1677–1681

[13] Melde K, Buettner H, Boschert W, Wolf HP, Ghisla S (1989): Mechanism of hypoglycaemic action of methylenecyclopropylglycine. *Biochem J*. 259: 921–924 2730593 10.1042/bj2590921 PMC1138607

[14] Isenberg SL, Carter MD, Graham LA, Mathews TP, Johnson D, Thomas JD et al (2015): Quantification of metabolites for assessing human exposure to soapberry toxins hypoglycin A and methylenecyclopropylglycine. *Chem*. *Res*. *Toxicol*. 28: 1753–1759 10.1021/acs.chemrestox.5b00205 26328472 PMC4592145

[15] Isenberg SL, Carter MD, Hayes SR, Graham LA, Johnson D, Methews TP et al (2016): Quantification of Toxins in Soapberry (Sapindaceae) Arils: Hypoglycin and Methylencyclopropylglycine. *J*. *Agri*. *Food*. *Chem*. 64: 5607–5613 10.1021/acs.jafc.6b02478 PMC5098216 27367968

[16] Sander J, Terhardt M, Sander S, Janzen N (2017) Quantification of Methylencyclopropyl Compounds and Acyl Conjugates by UPLC-MS/MS in the study of the biochemical effects of the Ingestion of canned Ackee (*Blighia sapida*) and Lychee (*Litchi chinensis*). *J of Agri*. *Food Chem* 65: 2603–2608 10.1021/acs.jafc.7b00224 28290200

[17] Tanaka K, Kean EA, Johnson B (1976): Jamaican Vomiting Sickness: Biochemical Investigationof two cases. *N*. *Engl*. *J*. *Med*. 295: 461–467 10.1056/NEJM197608262950901 940578

[18] Minakata H, Komura H, Tamura SY, Ohfune Y, Nakanishi K, Kada T (1985): Antimutagenic unusual amino acids from plants *Experientia* 41: 1622–1623 10.1007/BF01964840 3935481

[19] Melde K, Jackson S, Bartlett K, Sherratt HS, Ghisla S (1991) Metabolic consequences of methylenecyclopropylglycine poisoning in rats. *Biochem*. *J*. 274: 395–400 2006907 10.1042/bj2740395 PMC1150150

[20] Brown M, Bates RP, McGowan C, Cornell JA (1991): Influence of fruit maturity on the Hypoglycin A level in ackee (Blighia sapida) *J*.*Food Saf*. 12: 167–177

[21] Bowen-Forbes CS, Minott DA (2011): Tracking hypoglycins A and B over different maturity stages: Implications for detoxification of ackee (Blighia sapida K.D. Koenig Fruits) *J*. *Agric*. *Food Chem*. 59: 3869–3875 10.1021/jf104623c 21410289

[22] Li D, Agnihotri G, Dakoji S, Oh E, Lantz M, Liu H (1999): The toxicity of methylencyclopropylglycine: studies of the inhibitory effects of (methylencyclopropyl)formyl-CoA based on enzymes involved in fatty acid metabolism and the molecular basis of its inactivation of Enoyl-CoA hydratases. *J*. *Am*. *Chem*. *Soc*. 121: 3034–3042

[23] Dakoji S, Li D, Agnihotri G, Zhou H, Liu H (2001): Studies on the inactivation of bovine liver enoyl-CoA hydratase by (methylenecyclopropyl)formyl-CoA hydratase by (methylenecyclopropyl)formyl-CoA: Elucidiation of the inactivation mechanism and identification of cysteine-114 as the entrapped nucleophile. *J*. *Am*. *Chem*. *Soc*. 123: 9749–9759 11583536 10.1021/ja011226k

[24] Wu L, Lin S, Li D (2008): Comparative inhibition studies of enoyl-CoA hydratase 1 and enoyl-CoA hydratase 2 in long-chain fatty acid oxidation. *Org Lett*.10: 3355–3358 10.1021/ol801267e 18611036

[25] Tanaka K (1972): On the mode of action of hypoglycin A. 3. Isolation and identification of cis-4-decene-1,10-dioic, cis, cis-4,7-decadiene-1,10-dioic, cis-4-octene-1,8-dioic, glutaric, and adipic acids, N-(methylenecyclopropyl)acetylglycine, and N-isovalerylglycine from urine of hypoglycin A-treated rats. *J Biol Chem*. 247: 7465–7478 4636318

[26] Tanaka K, Ikeda Y (1990): Hypoglycin and Jamaican Vomiting Sickness *Prog*. *Clin*. *Biol*. *Res*. 321: 167–184 2183231

[27] Al-Bassam SS and Sherratt HSA (1981): The antagonism of the toxicity of hypoglycin by glycine. *Biochem*. *Pharamcol*. 30: 2817–2824 10.1016/0006-2952(81)90420-2 7317076

[28] Von Holt C, Chang J, von Holt M, Bohm H (1964): Metabolism and metabolic effects of hypoglycin *Biochem*. *Biophys*. *Acta*, *Gen*. *Subj*. 90: 611–613 10.1016/0304-4165(64)90242-9 14237871

[29] Blake OA, Bennink MR, and Jackson JC(2006): Ackee *(Blighia sapida)* hypoglycin A toxicity: Dose response assessment in laboratory rats. *Food Chem*. *Toxicol*. 44: 207–213 10.1016/j.fct.2005.07.002 16099087

[30] John TJ, Das M (2014): Acute encephelatitis syndrome in children in Muzaffarpur. *Curr*. *Sci*. 106: 1184–1185

[31] Bochnia M, Ziegler J, Sander J, Uhlig A, Schaefer S, Vollstedt S et al (2015): Hypoglycin A Content in Blood and Urine Discriminates Horses with Atypical Myopathy from Clinically Normal Horses Grazing on the Same Pasture. *PLoS One* 10(9): e0136785 10.1371/journal.pone.0136785 26378918 PMC4574941

[32] Bochnia M, Scheidemann W, Ziegler J, Sander J, Vollstedt S, Glatter M et al (2018): Predictive value of hypoglycin A and methylencyclopropalacetic acid conjugates in a horse with atypical myopathy in comparison to its cograzing partners. *Equine Vet Educ* 30:24–28 (10.1111/eve.12596).

[33] Żuraw A, Dietert K, Kühne l S, Sander J, Klopfleisch R(2016): Equine atypical myopathy caused by hypoglycin A intoxication associated with ingestion of sycamore maple tree seeds. *Equine Vet J*. 48: 418–21 10.1111/evj.12460 25970235

[34] Ghisla S, Melde K, Zeller HD, Boschert W (1990): Mechanisms of enzyme inhibition by hypoglycin, methylenecyclopropylglycine and their metabolites. *Prog Clin Biol Res*. 21: 185–192 2326291

[35] Chen KK, Anderson RC, McCowen MC, Harris PN (1957): Pharmacologic action of hypoglycin A and B. *J Pharmacol Exp Ther* 121: 272–285 13481850

[36] Agnihotri G, He S, Hong L, Dakoji S, Withers SG, Liu HW (2002): A revised mechanism for the inactivation of bovine liver enoyl-CoA hydratase by (methylenecyclopropyl) formyl-CoA based on unexpected results with the C114A mutant. 41: 1843–52 11827529 10.1021/bi0119363

[37] Paireau J, Tuan NH, Lefrancois R, Buckwalter MR, Nghia ND, Hien NT et al (2012) Litchi-associated acute encephalitis in children, Northern Vietnam, 2004–2009. Emerging Infect Dis. 18: 1817–1824 10.3201/eid1811.111761 23092599 PMC3559149

[38] Shrivastava A, Srikantiah P, Kumar A, Bhushan G, Goel G, Kumar S et al (2015) Outbreaks of unexplained neurologic illness-Muzaffarpur, India, 2013–2014 Morb. Mortal. Wkly. Rep., 64 (2015), pp. 49–53 PMC4584556 25632950

[39] Phan NT, Gouilh MA, Paireau J, Phuong L, Cheval J, Ngu ND et al (2018) Hypoglycemic toxins and enteroviruses as causes of outbreaks of acute encephalitis-like syndrome in children, Bac Giang Province, Northern Vietnam.Emerg Infect Dis.24:1435–1443. 10.3201/eid2408.171004 30014832 PMC6056107

